# AKT1 regulates UHRF1 protein stability and promotes the resistance to abiraterone in prostate cancer

**DOI:** 10.1038/s41389-022-00446-y

**Published:** 2023-01-02

**Authors:** Yongming Fu, Tuoyu Cao, Xiaorui Zou, Yubing Ye, Youhong Liu, Yuchong Peng, Tanggang Deng, Linglong Yin, Xiong Li

**Affiliations:** 1 Key Laboratory of Clinical Precision Pharmacy of Guangdong Higher Education Institutes, The First Affiliated Hospital, Guangdong Pharmaceutical University, Guangzhou, China; 2 Key Specialty of Clinical Pharmacy, The First Affiliated Hospital of Guangdong Pharmaceutical University, Guangzhou, China; 3 NMPA Key Laboratory for Technology Research and Evaluation of Pharmacovigilance, Guangdong Pharmaceutical University, Guangzhou, China; 4 Department of Oncology, Center for Molecular Medicine, Xiangya Hospital, Central South University, Changsha, China; 5 Hunan Key Laboratory of Molecular Radiation Oncology, Xiangya Hospital, Central South University, Changsha, China; 6 School of Clinical Pharmacy, Guangdong Pharmaceutical University, Guangzhou, China

**Keywords:** Cancer therapeutic resistance, Targeted therapies

## Abstract

Oncogenic activation of PI3K/AKT signaling pathway, together with epigenetic aberrations are the characters of castration-resistant prostate cancer (CRPC). UHRF1 as a key epigenetic regulator, plays a critical role in prostate cancer (PCa) development, and its expression is positively correlated with the degree of malignancy. In this present study we investigated the potential regulatory mechanism of AKT1 on UHRF1, and further validated the in vitro and in vivo anticancer efficacy of AKT phosphorylation inhibitor MK2206 in combination with abiraterone. Both UHRF1 and p-AKT aberrantly overexpressed in the abiraterone-resistant PCa cells. Further studies revealed that AKT1 protein interacts with UHRF1, and AKT1 directly phosphorylates UHRF1 via the site Thr-210. MK2206 induced UHRF1 protein degradation by inhibiting AKT1-induced UHRF1 phosphorylation, and then reduced the interaction between UHRF1 and deubiquitinase USP7, while promoted the interaction between UHRF1 and E3 ubiquitin protein ligase BTRC. MK2206 significantly promoted the sensitivity of abiraterone-refractory PCa cells and xenografts to abiraterone by decreasing UHRF1 protein level, and reversed the phenotype of NEPC, evently induced cellular senescence and cell apoptosis. Altogether, our present study for the first time revealed a novel molecular mechanism of abiraterone resistance through PI3K/AKT-UHRF1 pathway, and provided a novel therapeutic modality by targeting PI3K/AKT1 to promote the drug sensitivity of abiraterone in PCa patients.

## Introduction

Prostate cancer (PCa) is the most common cancer type among man except for skin cancer, which has nearly 1.4 million new cases and almost 375,000 reported deaths worldwide in 2020 [1]. In China, the incidence rate of PCa was ~10.23/100,000, taking the sixth incidence of male malignancy, and the crude mortality of PCa was 4.36/100,000 in 2015 [2].

The initiation and progression of PCa overwhelmingly depends on androgen/androgen receptor (AR), thereby androgen deprivation therapy (ADT) has become the first-line therapeutic modality for men with advanced PCa. However, ADT resistance develops within 18–24 months, and then the disease develops to castration-resistant PCa (CRPC) [3]. The 5-year survival rate of CRPC patients is only 25–33%, and ≥84% of CRPC patients develops metastasis at diagnosis [4, 5]. The second generation AR Antagonists, such as abiraterone and enzalutamide have been approved to treat metastatic CRPC (mCRPC), and prolonged overall survival (OS) of mCRPC patients [6, 7]. However, drug resistance inevitably develops, and the life quality of patients is pretty poor. Several underlying mechanisms of ADT resistance, such as AR amplification, mutation, AR splice variants, abnormal expression of AR co-activators, or co-repressors, glucocorticoid receptor upregulation have been proposed [8, 9].

Phosphatidylinositide-3-kinase (PI3K)/AKT pathway promotes the initiation and progression of PCa by promoting cell proliferation, invasion, autophagy, and metabolism [10–12]. The PI3K/AKT pathway activates the genomic and non-genomic AR and other signaling cascades [13, 14]. Furthermore, the activation of PI3K/AKT pathway results in drug resistance by elevating anti-apoptotic molecule Bcl-2 [15]. Several molecular inhibitors of PI3K/AKT pathway have been developed [16]. AKT inhibitor ipatasertib in combination with abiraterone significantly improved the survival of mCRPC patients [17], suggesting that the aberration of PI3K/AKT signaling may be one of crucial factors of abiraterone resistance.

Ubiquitin-like containing PHD Ring Finger 1 (UHRF1) is a key epigenetic regulator and plays key roles in bridging DNA methylation and histone modfication [18]. UHRF1 has been involved in a series of cellular activities, such as DNA replication, DNA damage repair, etc [19–21]. UHRF1 is aberrantly overexpressed in many cancer types, and its expression level is positively correlated with the degree of malignancy [22–24]. The knockdown of UHRF1 retarded cell proliferation, induced cell apoptosis, and cell metabolism [25–27]. Therefore, it has been considered as a potential therapeutic target of PCa. UHRF1 is regulated by post-translational modifications such as ubiquitination, methylation, and phosphorylation [28–30]. However, whether PI3K/AKT regulates the post-translational modification of UHRF1 remains unclear.

In this present study, we generated two pairs of abiraterone-refractory or parental PCa cell lines LNCaP/LNCaP-R and CWR22Rv1/CWR22Rv1-R. We firstly observed that higher expression levels of UHRF1 and p-AKT in the abiraterone-resistant PCa cells than the parental cells. In addition, AKT1 phosphorylated UHRF1 and sustained its protein stability. MK2206 as a AKT1 phosphorylation inhibitor, reduced the protein interaction between AKT1 and UHRF1, thereby promoted UHRF1 protein degradation. MK2206 significantly promoted the sensitivity of abiraterone in the abiraterone-resistant PCa cells and xenografts, and reversed the phenotype of NEPC, evently induced cellular senescence and cell apoptosis. This study provided a novel therapeutic modality in which targeting AKT1 by small molecule inhibitor promoted drug sensitivity of abiraterone in PCa patients.

## Materals and methods

### Cell lines and cell culture

PCa cells LNCaP and VCaP and normal prostate epithelial cells RWPE-1 were purchased from the American Type Culture Collection (ATCC). C4-2 and CWR22Rv1 cells were the gifts from Dr. Chinghai Kao in Department of Urology, Indiana University School of Medicine. HEK293T cells were obtained from Center for Molecular Medicine of Xiangya Hospital, Central South University. LNCaP, VCaP, C4-2, and CWR22Rv1 were cultured in RPMI-1640 medium supplied with 10% fetal bovine serum (ExCellBio, Shanghai, China). HEK293T were cultured in DMEM supplied with 10% fetal bovine serum. RWPE-1 was cultured in defined Keratinocyte-SFM (1×) liquid (Invitrogen, Carlsbad, CA, USA). All cells were incubated at 37 °C with 5% CO2.

### Generation of LNCaP-R and CWR22RV1-R cell lines

LNCaP or CWR22Rv1 cells were initially cultured in the media containing 20 uM abiraterone, and the living cells were cultured with the gradually increased drug concentrations for continuous 6 months. The IC50 values of cells response to abiraterone were identified by crystal violet assays. The LNCaP or CWR22Rv1 cells, whose IC50 values of abiraterone are over 50 uM were regarded as the abiraterone-resistant LNCaP-R and CWR22Rv1-R cells.

### Antibodies and reagents

Anti-UHRF1 (A2343), Anti-β-Tubulin (A12289), Anti-NCAM1 (A7913), Anti-Synaptophysin (A6344), Anti-USP7 (A13564), Anti-BTRC (A18232) were purchased from ABclonal (Wuhan,HuBei, China). Anti-Cleaved-PARP, Anti-AKT (phospho S473) were purchased from Abcam (Cambirdge,MA,USA). Anti- Akt (pan) (#4685), Anti-HA Tag (#3724), Anti-p21(#2947), Anti-Phospho-AKT (Thr308) (#13038), Anti-Normal Rabbit IgG (#2729) were purchased from Cell Signaling Technology(Danvers, MA, USA), Anti-AKT1 antibody [HL1145] (#GTX636416) was purchased from GenTex (Irvine, CA, USA), Anti-His Tag(A00186) was purchased from GenScript (Nanjing, Jiangsu, China), p-Thr(H-2) was purchased from Santa Cruze Biotechnology(Shanghai, China). Monoclonal ANTI-Flag® M2 antibody (F1804) and Normal Mouse IgG (12-371) were purchased from Merck (Darmstadt, Germany).

Cycloheximide (HY-12320), MK2206 (HY-108232), and Protein A/G Magnetic Beads (HY-K0202) were purchased from MedChemExpress LLC. (Shanghai, China). MG132 (T2154) was purchased from Topscience Co. Ltd. (Shanghai, China). Lipo6000™ Transfection Reagent(C0526), a cationic liposome for transfection of HEK293T cells, was purchased from Beyotime (Shanghai, China).

### Plasmids and primers

The wild-type UHRF1 and UHRF1-T210A mutant were cloned into pcDNA3.1/His or pGEX4T-1 vectors, and pcDNA3.1-Flag-myr-AKT1 was purchased from MiaoLing Plasmid Sharing Platform (Wuhan, Hubei, China). The sequences of primers for PCR in this study are as follows. UHRF1(Forward: 5’GACAAGCAGCTCATGTGCGATG3’; and reverse: 5’ AGTACCACCTCGCTGGCATCAT3’) and ACTIN(Forward: 5’ CACCATTGGCAATGAGCGGTTC3’, and reverse: 5’ AGGTCTTTGCGGATGTCCACGT3’).

### RNA extraction and qRT-PCR

Total RNA was exacted by using RNAiso PLUS following the manufacturer’s instructions. Total RNA (1 ug) was used for synthesizing the first strand cDNA by using Revert Aid First Strand cDNA Synthesis Kit (Thermo Scientific™). 2×SYBR Green qPCR Master Mix (Bimake, Houston, TX) were used for the qPCR. The relative mRNA expression levels were normalized to ACTIN. The statistical difference of the results was analyzed by Student’s *t*-test, and the significance of data was determined by *p*-value <0.05.

### Western blotting

Cells were washed with cold PBS three times and lysed with cell lysis buffer (Beyotime) for western blotting and IP assays. Cell lysates were separated by SDS-PAGE and then transferred to PVDF membranes (Millipore, Burlington, MA. USA), followed by blocking in 5% milk for 1 h at room temperature. The membranes were washed with TBS containing 1% Tween 20 for three times, and incubated with primary antibodies overnight at 4 °C. After incubating with the second antibodies, the membranes were exposed to chemiluminescence substrate, and the signals were detected by using Chemiluminescence Image Analysis System (Clontech, Mountain View, USA).

### Co-immunoprecipitation assay

1 × 10^6^ HEK293T cells were co-transfected with pcDNA3.1/His-UHRF1 and pcDNA3.1-Flag-myr-AKT1. The transfected cells were washed with cold PBS for three times and lysed in cell lysis buffer 48 h after transfection. The cell extracts were incubated with 1 ug Anti-His Tag (A00186, GenScript, Nanjing, Jiangsu, China) or 1 ug Anti-Flag Tag (F1804) at 4 °C overnight, followed by incubating with 20 ul Protein A/G Magnetic Beads at 4 °C for one hour. The beads coupled with immunoprecipitates were washed five times with cell lysis buffer, and then harvested for western blotting.

### Ubiquitination detection assay

HEK293T cells were co-transfected with pcDNA3.1/His-UHRF1, pcDNA3.1- Flag-myr-AKT1, and pcDNA3.1-HA-Ub for 48 h, and then were treated with 50 uM MG132 plus DMSO or 10 uM MK2206 for 8 h, respectively. Then cells were lysed in RIPA cell lysis buffer and the extraction were incubated with 1 ug Anti-His Tag(A00186, GenScript) at 4 °C overnight on a rotating shaker. Protein A/G Magnetic Beads (20 ul) were added and incubated for 1 h. The immunoprecipitates were used for western blotting analysis.

### Recombinant protein purification

The plasmids expressing GST-UHRF1-WT and GST-UHRF1-T210A were constructed in pGEX4T-1 vector, and the recombinant proteins were generated and purified following the manufacturers’ manuals (Sangon, Shanghai, china). Briefly, the plasmids pGEX4T-1-UHRF1-WT or pGEX4T-1-UHRF1-T210A were separately transformed to Bacterial BL21 cells for 12 h, and 50 mg/ml IPTG was added to induce the expression of GST-UHRF1-WT and GST-UHRF1-T210A at 20 °C for 16 h. Next, the BL21 cells were collected, and GST tagged UHRF1-WT and UHRF1-T210A proteins were purified by using GST-Sefinose (TM) Resin 4FF (Sangon). The purity of GST-UHRF1-WT or GST-UHRF1-T210A was detected by Coomassie staining after SDS-PAGE.

### In vitro kinase assay

pcDNA3.1-Flag-myr-AKT1 was transfected to HEK-293T cells, and the myr-AKT1 proteins firstly were immunoprecipitated with the anti-Flag antibody. The myr-AKT1 immunoprecipitates were resuspended in 500 μl 1× kinase buffer (#9802, Cell Signaling Technology) including 500 μM ATP (#9804, Cell Signaling Technology), and then co-cultured with 1ug recombinant proteins GST-UHRF1-WT or GST-UHRF1-T210A or negative control at 30 °C for 30 min, and then the reaction was stopped by adding SDS sample buffer at 95 °C for 5 min. The sample were used to western blotting.

### Cell viability assay

RWPE-1, LNCaP, C4-2, CWR22Rv1, VCaP, LNCaP-R, and CWR22Rv1-R were seeded in 24-well cell culture plates, and treated with the indicated concentrations of abiraterone and MK2206 alone or in combination for 7 days. The cells were fixed with 4% paraformaldehyde and stained with 0.1% crystal violet solution for 15 min. After removing the unbound crystalline violet, the plates were washed with ddH_2_O and dried at 37 ℃ for 1 h. The cells were lysed on a shake table overnight by 1% SDS. Cellular viability was assessed by measuring the absorbance at 590 nm with VICTOR Nivo microplate reader.

### Cell apoptosis assay

Apoptotic cells were quantified by using the Annexin V/FITC Apoptosis Detection Kit (BD Biosience, Shanghai, China) by following the manufacturer’s instruction. PCa cells were treated with the indicated concentrations of abiraterone or MK2206 alone or in combination for 2 days, then the cells were harvested by using EDTA free trypsin and centrifuged at 1000 rpm for 3 min. Afterward, Cells were washed with PBS three times and incubated with 100 ul of the 1× binding buffer including 5 ul PI and 5 ul Annexin-V for 15 min. The positive Annexin-V/PI staining cells were detected by BD FACSAria II flow cytometer.

### In vivo study

The animal experiments were approved by the Institutional Animal Care and Use Committees(IACUCs) of The First Affiliated Hospital, Guangdong Pharmaceutical University according to the corresponding guidelines. Firstly, the xenograft models were induced by subcutaneously injecting 5 × 10^6^ CWR22Rv1 cells into the left axillary of 4–5-week-old male BALB/c-nude mice (*n* = 20). After the volume of the xenografts reached 100 mm^3^, the animals were randomly divided into four groups, and then treated with vehicle (control), abiraterone (100 mg/kg.d, i.g., every 3 days), MK2206 (70 mg/kg.d, i.g., every 3 days), or abiraterone (100 mg/kg.d, i.g., every 3 days) plus MK2206 (70 mg/kg.d, i.g., every 3 days) for 15 days. The tumor volume was calculated by the following equation: (L × W^2^)/2 (length (L) and width (W)).The body weight and tumor volume of mice were measured every 3 days, and the tumor growth curve and mouse body weight growth curve were plotted. At the endpoint of experiments, the tumors were dissected, and the weights of tumor nodes and animal bodies were recorded.

### Statistical analysis

All statistical analyses were performed by using SPSS 23.0 software. Student’s two-tailed *t*-test was used to analyze the statistical difference between two sets of data. One-way ANOVA was used for statistical analysis of difference of multiple groups. Results were represented as mean ± SD. All tests were two-tailed, *P*-values <0.05 were considered statistically significant (*P < 0.05, **P < 0.01, and ***P < 0.001).

## Results

### The levels of UHRF1 and p-AKT elevated in abiraterone-refractory PCa cell lines and NEPC tumor specimens

**Fig. 1:**
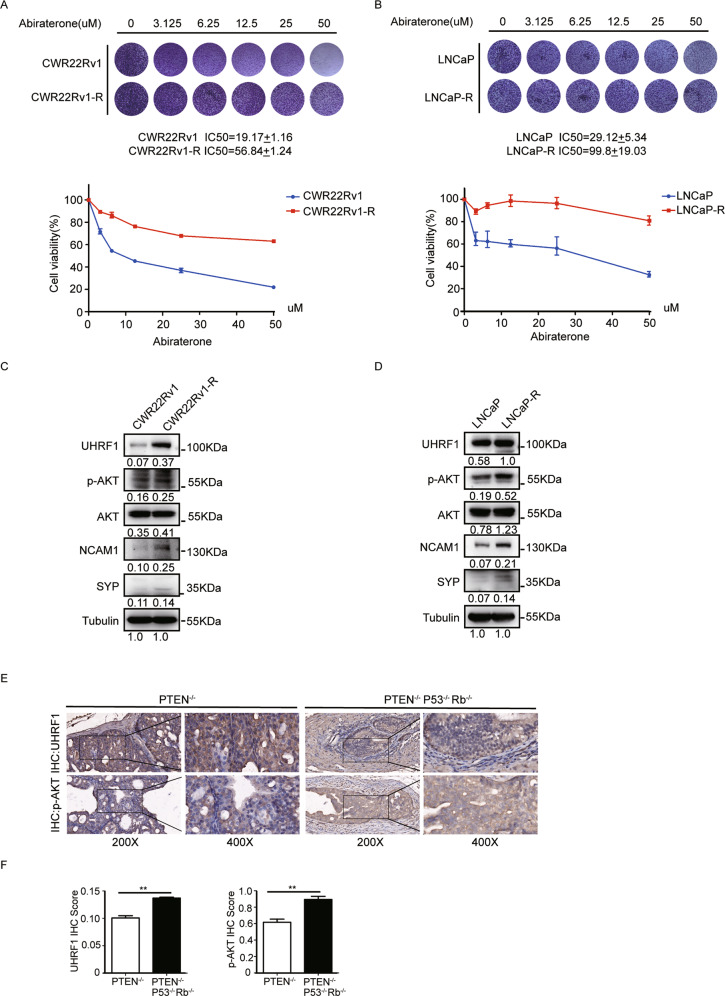
The levels of p-AKT and UHRF1 elevated in the abiraterone-refractory PCa cells. **A**, **B** The parental or abiraterone-refractory CWR22Rv1 (CWR22Rv1-R) or LNCaP (LNCaP-R) cells were treated with stepwise concentrations of abiraterone, and the IC50 values of abiraterone were calculated. **C**, **D** The protein levels of p-AKT, AKT, UHRF1, NCAM1, and SYP in the parental or abiraterone-refractory PCa cells were assessed by western blotting. **E**, **F** The expression level of UHRF1 and p-AKT were compared in the PCa tumor specimens from prostate specific PTEN^−^/P53^−^/Rb1^−^ or PTEN^*−*^ gene knockout mice. The presented results were representative of experiments repeated at least three times. Data was represented as mean ± SD. *P < 0.05, **P < 0.01, ***P < 0.001.

It has been reported that the expression of UHRF1 steadily increased with the elevation of Gleason grade of prostate cancer (PCa) [24]. Furthermore, the overexpression of UHRF1 is closely correlated with drug resistance of PCa [31]. We spectulated UHRF1 aberrant overexpression may be one of typical characteristics of the abiraterone resistance. We first established two pairs of abiraterone-refractory PCa cell lines (CWR22Rv1/CWR22Rv1-R and LNCaP/LNCaP-R) by exposing the parental cells with steadily-increasing drug doses for 6 months. We then examined the sensitivity of these two pairs of PCa cell lines to abiraterone by using crystal violet assays. Abiraterone sensitive or refractory CWR22Rv1 or LNCaP cells were treated with stepwise concentrations of abiraterone, and the IC50 values were calculated. The data showed that abiraterone inhibited the proliferation of PCa cells in a dose-dependent manner. The IC50 values increased 3–4 folds in the abiraterone-refractory cell lines compared to the parental cell lines (Fig. 1A and B). Additionally, it has well known that PCa develops to neuroendocrine prostate cancer (NEPC), which is the most deadly subtype of PCa when PCa develops abiraterone resistance, and expressed typical NEPC markers including NCAM1 (CD56) and SYP [32, 33]. We next compared the expression of UHRF1, p-AKT, AKT, NCAM1, and SYP in the abiraterone-refractory to parental PCa cell lines. UHRF1, p-AKT, NCAM1, and SYP significantly elevated in the abiraterone-refractory PCa cell lines (Fig. 1C and D). We further assessed the protein levels of UHRF1 and p-AKT in PCa tumor specimens of prostate specific PTEN^−^/P53^−^/Rb1^−^ or PTEN^−^ gene knockout mice by IHC analysis (*n* = 3). In consistent to the published data [34], the PCa tumors of PTEN^−^/P53^−^/Rb1^−^ gene knockout mice developed to the NEPC phenotype. and the levels of UHRF1 protein and p-AKT were significantly higher in NEPC than PCa tumors of PTEN^*−*^ gene knockout mice (Fig. 1E and F). Collectively, UHRF1 and p-AKT remarkably elevatated when PCa develops abiraterone resistance and NEPC transdifferentiation. Therapeutics targeting UHRF1 or p-AKT may be an effective strategy to overcome abiraterone resistance.

### AKT1 inhibitor MK2206 sensitized abiraterone-refractory PCa cells to the treatment of abiraterone

**Fig. 2:**
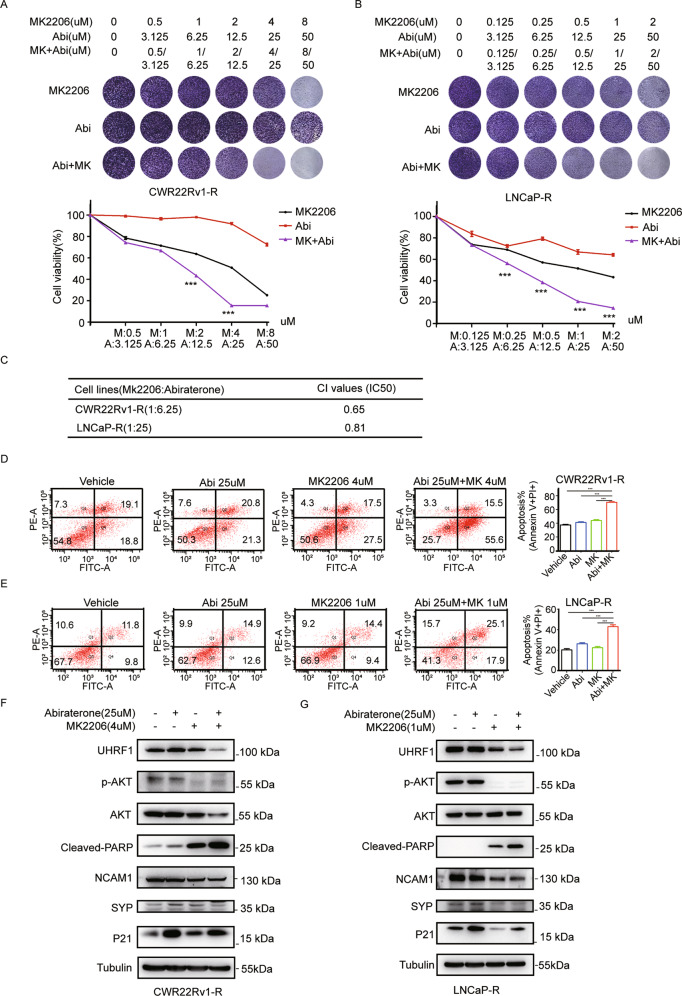
AKT1 inhibitor MK2206 sensitized abiraterone-refractory PCa cells to the treatment of abiraterone. **A**, **B** CWR22Rv1-R (**A**) and LNCaP-R cells (**B**) were seeded in 24-well plates, and then treated with stepwise concentrations of abiraterone or MK2206 alone or in combination for 7 days. The living cells were measured by crystal violet assay. **C** The CI values of abiraterone and MK2206 in CWR22Rv1-R or LNCaP-R cells. **D**, **E** CWR22Rv1-R (**D**) and LNCaP-R cells (**E**) were treated with abiraterone or MK2206 alone or in combination for 48 h, and the apoptotic cells were stained with Annexin V/PI, and analyzed by flow cytometry. **F**, **G** CWR22Rv1-R (**F**) and LNCaP-R cells (**G**) were treated with abiraterone or MK2206 alone or in combination for 48 h. UHRF1, AKT, the phosphorylated AKT, and cleaved-PARP were assessed by western blotting. The presented results were representative of experiments repeated at least three times. Data was represented as mean ± SD. *P < 0.05, **P < 0.01, ***P < 0.001.

UHRF1 plays a vital role in PCa by regulating cell cycle, proliferation, apoptosis, and metabolism. In addition, the overexpression of UHRF1 resulted in drug resistance [35]. We speculated that targeting AKT1-UHRF1 axis may restore the sensitivity of abiraterone-refractory cell lines to abiraterone treatment. We treated the abiraterone-refractory CWR22Rv1-R or LNCaP-R cell lines with different concentration combinations of abiraterone and MK2206 for 7 days, and the living cells were stained with crystal violet dye. As shown in the Fig. 2A and B, the curves of cell proliferation revealed that abiraterone or MK2206 inhibited cell proliferation in a dose-dependent manner. Furthermore, the combination of abiraterone and MK2206 demonstrated synergistic inhibitory effect on cell proliferation in CWR22Rv1-R or LNCaP-R cells. The CI values of MK2206 and abiraterone in CWR22Rv1-R cells (1:6.25) and LNCaP-R cells (1:25) are 0.65 or 0.81, respectively (Fig. 2C). Similarly, the apoptosis-detection assay revealed that abiraterone or MK2206 induced cell apoptosis, and the combination of abiraterone and MK2206 synergistically induced cell apoptosis in CWR22Rv1-R or LNCaP-R cells (Fig. 2D and E). Mechanistically, abiraterone or MK2206 decreased the protein levels of UHRF1 and p-AKT, and the combination of abiraterone and MK2206 further decreased the protein levels of UHRF1 and p-AKT(Fig. 2F and G). In addition, the combination of abiraterone and MK2206 significantly elevated the level of the cleaved-PARP (Fig. 2F and G), which is consistent to the results of apoptosis-detection assay (Fig. 2D and E). These results suggested that inhibition of AKT1-UHRF1 axis promoted the sensitivity of abiraterone in the abiraterone-refractory cell lines.

### AKT phosphorylation inhibitor promotes the degradation of UHRF1 protein through ubiquitin-proteasome pathway

**Fig. 3:**
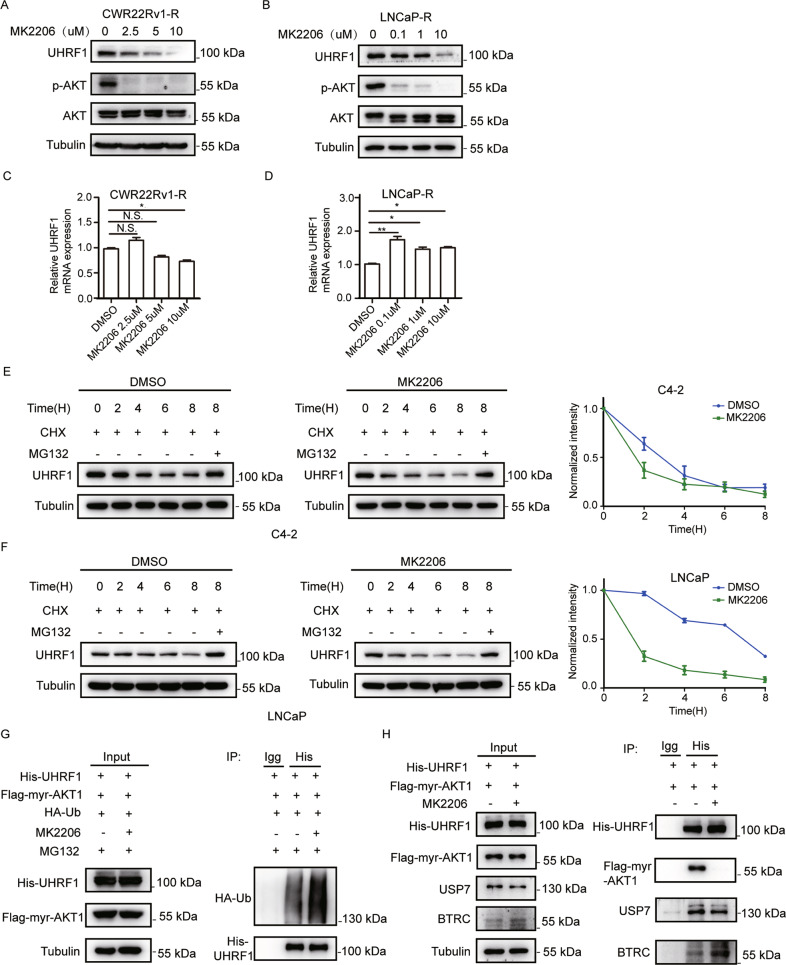
AKT inhibition promotes the degradation of UHRF1 protein through ubiquitin-proteasome pathway. **A**, **B** CWR22Rv1-R (**A**) or LNCaP-R cells (**B**) were treated with stepwise concentrations of MK2206 for 24 h, and the protein levels of UHRF1 or p-AKT were assessed by western blotting. **C**, **D** The mRNA levels of UHRF1 were measured by qRT-PCR. **E**, **F** C4-2 (**E**) or LNCaP (**F**) cells were treated with 10 μM MK2206, together with 50 uM cycloheximide (CHX) for the indicated time, or were treated with 10 μM MK2206 plus 50 uM CHX and 50 mM MG132 for 8 h. UHRF1 protein levels were assessed by western blotting and the bands were quantified by image-J software. **G** HEK-293T cells were co-transfected with the plasmids encoding His-UHRF1, Flag-myr-AKT1, and HA-ubiquitin for 48 h, and then treated with 50 μM MG132 plus 10 μM MK2206 for additional 8 h. UHRF1 protein was immunoprecipitated with ant-His antibody, and the ubiquitinated-UHRF1 were assessed with anti-HA antibody. **H** HEK-293T cells were co-transfected with the plasmids encoding His-UHRF1 or Flag-myr-AKT1, and then treated with 10 μM MK2206 for 24 h. UHRF1 protein was immunoprecipitated with anti-His antibody, and deubiquitinase USP7 or E3 ubiquitin protein ligase BTRC were measured by western blotting. The presented results were representative of experiments repeated at least three times. Data was represented as mean ± SD. *P < 0.05, **P < 0.01, ***P < 0.001.

No UHRF1-targeted inhibitor has been testing in clinic trials, while several AKT phosphorylation inhibitors have been developed, and demonstrated significant anticancer efficacy in the pre-clinical or clinical trials [36, 37]. We proposed whether AKT phosphorylation inhibitor destroyed the protein stability of UHRF1. CWR22Rv1-R or LNCaP-R was treated with AKT kinase inhibitor MK2206 for 24 h. The protein or mRNA level of UHRF1 were assessed by western blotting or qRT-PCR, respectively. As shown in Fig. 3A and B, MK2206 significantly decreased the levels of UHRF1 protein and p-AKT in a dose-dependent manner. However, MK2206 did not demonstrate a consistent effect on UHRF1 mRNA level in LNCaP-R or CWR22Rv1-R cells. In LNCaP-R cells at least the UHRF1 mRNA expression significantly increased, while in the CWR22Rv1-R cells the change in UHRF1 mRNA levels was MK2206 concentration-dependent. (Fig. 3C and D). Similarly, Knockdown of AKT1 significantly decreased UHRF1 protein levels, but did not make a consistent effect on UHRF1 mRNA levels in CWR22Rv1-R and LNCaP-R cells (Supplementary Fig. 1A, 1B, 1C, and 1D). These data suggested that AKT phosphorylation inhibitors promoted UHRF1 protein degradation.

We next added cycloheximide (CHX) to inhibit the synthesis of protein, or/and proteasome inhibitor MG132 when treated PCa cells with MK2206, and monitored the protein stability of UHRF1. As expected, MK2206 remarkably accelerated the protein degradation of UHRF1, and this degradation was reversed by MG132 (Fig. 3E and F). In LNCaP cells, knockdown of AKT1 significantly promoted protein degradation of UHRF1 (Supplementary Fig. 1E). These data suggested that AKT inhibitor promoted UHRF1 protein degradation through ubiquitin-proteasome pathway. We further validated the data by the in vitro ubiquitination assay. These plasmids encoding HA-Ub, His-UHRF1 and Flag-myr-AKT1 were co-transfected to HEK-293T cells, and then were treated with MK2206 for 8 h. The data showed that AKT phosphorylation inhibition or AKT1 knockdown significantly promoted the ubiquitination of UHRF1 protein (Fig. 3G and Supplementary Fig. 1F), thereby resulting in protein degradation. The data suggested that AKT phosphorylation is required for the maintenance of UHRF1 protein stability. It is well known that protein stability is maintained by promoting the interaction of deubiquitinase or/and decreasing the interaction of E3 ubiquitin protein ligase. It has been reported that the deubiquitinase USP7 and the E3 ubiquitin protein ligase BTRC play critical roles in the maintenance of UHRF1 protein stability [30, 38]. We then examined the effects of AKT phosphorylation inhibition on the protein interactions between UHRF1 and USP7, or UHRF1 and BTRC by co-immunoprecipitation. The results showed that AKT inhibition or AKT1 knockdown remarkably decreased the protein interaction between UHRF1 and USP7, while increased the interaction between UHRF1 and BTRC (Fig. 3H and Supplementary Fig. 1G). These results suggested that AKT inhibition promoted UHRF1 protein degradation by promoting BTRC-induced ubiquitination and decreasing USP7-mediated deubiquitynation.

### AKT induces UHRF1 phosphorylation at the threonine 210 residue, and sustains UHRF1 protein stability through deubiquitinase USP7

**Fig. 4:**
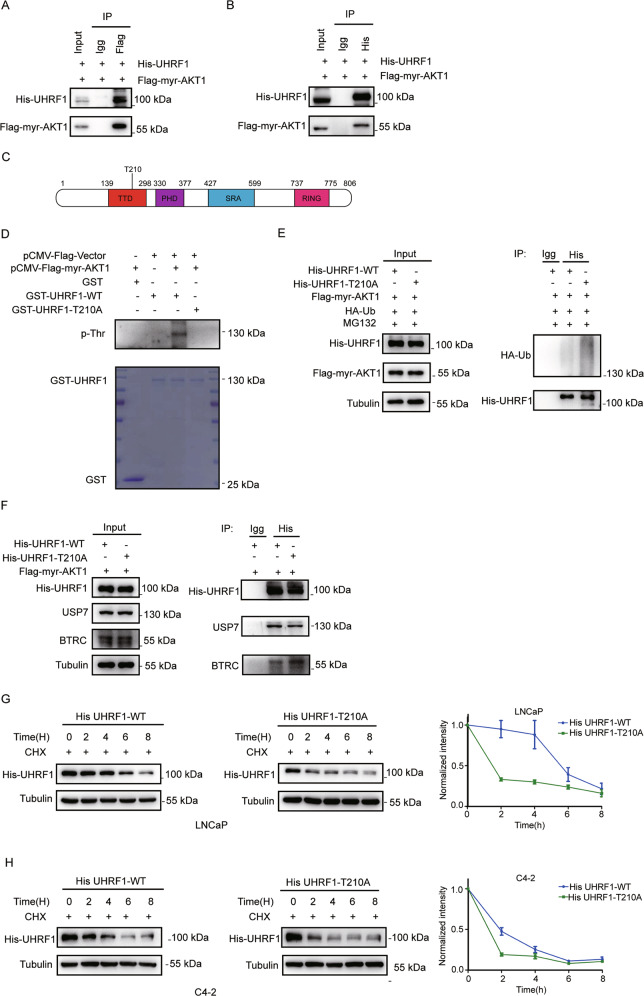
AKT induces UHRF1 phosphorylation via the threonine 210 residue, and sustains UHRF1 protein stability via binding deubiquitinase USP7. **A**, **B** HEK-293T cells were co-transfected with the plasmids encoding His-UHRF1 and Flag-myr-AKT1. AKT, or UHRF1 protein was immunoprecipitated by using Anti-Flag antibody (**A**) or Ant-His antibody (**B**), The AKT or UHRF1 protein was assessed by western blotting. **C** An AKT consensus motif (RXRXXS/T) was identified in the TTD domain of UHRF1 protein. **D** The wild-type or mutant of UHRF1 (GST-UHRF1-WT or T210A) or FLAG-myr-AKT1 was constructed, and the recombinant proteins were purified. A in vitro kinase assay was performed by incubating the purified proteins with the magnetic beads, and the phosphorylated UHRF1 was detected by immunoblotting with anti-threonine phosphorylation antibody. **E** HEK-293T cells were co-transfected with the plasmids encoding His-UHRF1(WT or T210A), Flag-myr-AKT1 and HA-ubiquitin for 48 h, and then treated with 50 μM MG132 for 8 h. UHRF1 protein was immunoprecipitated by using anti-His antibody, and the ubiquitinated-UHRF1 were detected with anti-HA antibody by western blotting. **F** HEK-293T cells were co-transfected with the plasmids encoding His-UHRF1(WT or T210A), Flag-myr-AKT1 for 48 h. UHRF1 protein was immunoprecipitated with anti-His antibody, and UHRF1 or BTRC protein was detected by western blotting. **G**, **H** LNCaP and C4-2 cells were transfected with the plasmids encoding His-UHRF1-WT or T210A for 48 h, and then treated with 50 uM cycloheximide (CHX) for the indicated time. The protein level of UHRF1 were detected with anti-His antibody by western blotting. The presented results were representative of experiments repeated at least three times. Data was represented as mean ± SD. *P < 0.05, **P < 0.01, ***P < 0.001.

It has been reported that UHRF1 can be phosphorylated by many kinases such as CDK1 or CDK2 [38, 39], but it is still unclear whether UHRF1 may be directly phosphorylated by AKT kinase. To validate that AKT kinase is required to sustain the protein stability of UHRF1, we first tested whether AKT protein interacts with UHRF1 by co-immunoprecipitation. The plasmids encoding His-UHRF1 and Flag-myr-AKT1 were co-transfected to HEK-293T cells. UHRF1 or AKT was immunoprecipitated with anti-His or anti-Flag antibody from the cell lysates, and the protein of AKT or UHRF1 was detected with anti-Flag or anti-His antibody by western blotting. As shown in Fig. 4A and B, both AKT and UHRF1 were detected in their individual immunoprecipitated complexes, but not in the isotype-matched negative control IgG complexes. The data validated that UHRF1 is a novel interactive substrate of AKT1.

By analyzing the amino acid sequence of UHRF1, we identified an AKT consensus motif (RXRXXS/T) at the threonine 210 residue (T210) in the TTD domain of UHRF1 protein, which may be a potential phosphorylation site of AKT1 (Fig. 4C). We performed an in vitro phosphorylation assay to verify the prediction by using the purified AKT1 and wild-type or mutated UHRF1 in which threonine 210 was substituted by alanine (T210A). The data showed that the wild-type UHRF1 may be phosphorylated by AKT1, while the T210A-mutated UHRF1 could not be phosphorylated (Fig. 4D). Furthermore, we explored whether the mutation of T210 phosphorylation site changed the protein ubiquitination of UHRF1 by the in vitro ubiquitination assay. These plasmids encoding HA-Ub, His-UHRF1-WT/His-UHRF1-T210A and FLAG-myr-AKT1 were co-transfected to HEK-293T cells. The data showed that the mutation of AKT phosphorylation sites of UHRF1(T210A) significantly promoted the ubiquitination of UHRF1 protein (Fig. 4E). Furthermore, the T210A mutation significantly decreased the protein interaction between UHRF1 and USP7, while promoted the protein interaction between UHRF1 and BTRC (Fig. 4F). We further found that the T210A mutation markedly promoted UHRF1 protein degradation in a dose-dependent manner in C4-2 and LNCaP cells.(Fig. 4G and H). These results suggested that AKT1 induced the phosphorylation of UHRF1, and promoted its protein stability by inhibiting UHRF1 protein degradation through ubiquitination-proteasome pathway.

### The combination of abiraterone and AKT inhibitor MK2206 selectively and synergistically inhibited the proliferation of PCa cells in vitro and xenografts growth in vivo

**Fig. 5:**
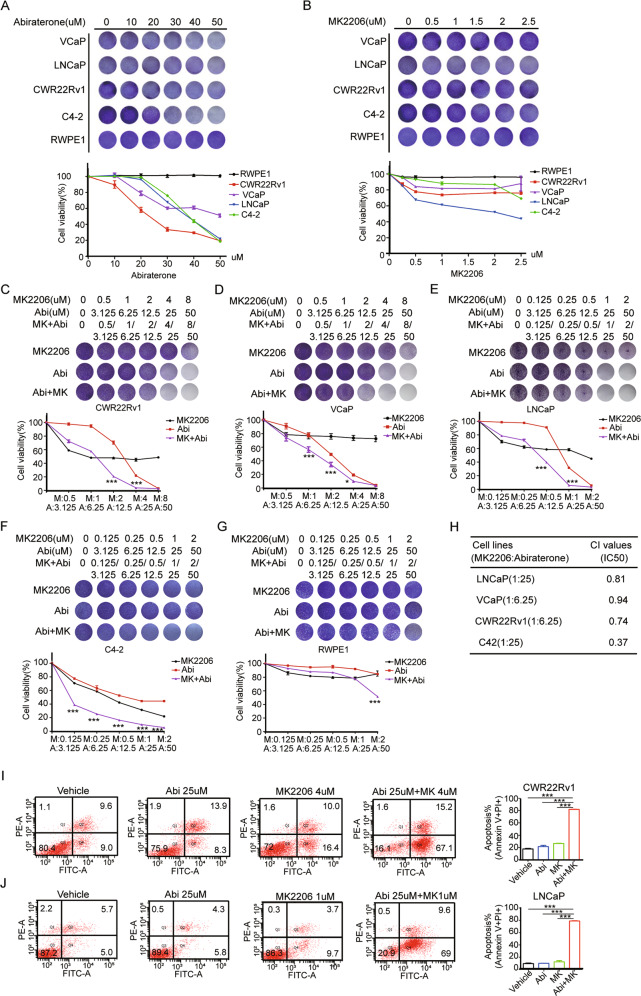
The combination of abiraterone and AKT inhibitor MK2206 selectively and synergistically inhibited the proliferation of PCa cells in vitro. **A**, **B** PCa cell lines VCaP, CWR22Rv1, LNCaP, C4-2, or normal prostate epithelial cells RWPE-1 were seeded in 24-well plates, and then treated with stepwise concentrations of abiraterone or MK2206 for 7 days. The living cells were measured by crystal violet assay. **C**–**G** VCaP, CWR22Rv1, LNCaP, C4-2, or RWPE-1 cells were seeded in 24-well plates, and then treated with stepwise concentrations of abiraterone or MK2206 alone or in combination for 7 days. The living cells were measured by crystal violet assay. **H** the CI (combination index) values of abiraterone and MK2206 were calculated in PCa cells. **I**, **J** CWR22Rv1 (**I**), LNCaP cells (**J**) were treated with abiraterone or MK2206 alone or in combination for 48 h, and the apoptotic cells were stained with Annexin V/PI, and analyzed by flow cytometry. The presented results were representative of experiments repeated at least three times. Data was represented as mean ± SD. *P < 0.05, **P < 0.01, ***P < 0.001.

We investigated the effect of abiraterone or/and MK2206 on the cell proliferation of PCa or normal cells, respectively, by the crystal violet assays. The data showed that abiraterone or MK2206 selectively repressed the proliferation of PCa cells in a dose-dependent manner, but no detectable toxicity on RWPE-1 cells (Fig. 5A and B). To examine whether the combination of abiraterone and MK2206 induced a synergistic killing effect on PCa cells, VCaP, CWR22Rv1, LNCaP, and C4-2 and non-malignant RWPE-1 cells were treated with abiraterone or MK2206 alone or in combination, and the cell viability was determined by crystal violet assays. The results showed that abiraterone and MK2206 synergistically inhibited the proliferation of PCa cells, but little impact on normal cells (Fig. 5C–G). The CI values of MK2206 and abiraterone in LNCaP cells (1:25), VCaP cells (1:6.25),CWR22Rv1 cells (1:6.25), and C4-2 cells(1:25) are 0.81, 0.94, 0.74, or 0.37, respectively (Fig. 5H). Furthermore, the combination of abiraterone and MK2206 induced more apoptosis of PCa cells than abiraterone or MK2206 alone (Fig. 5I and J). These results suggested that the combination of abiraterone and MK2206 selectively and synergistically inhibited the in vitro proliferation of PCa cells.

**Fig. 6:**
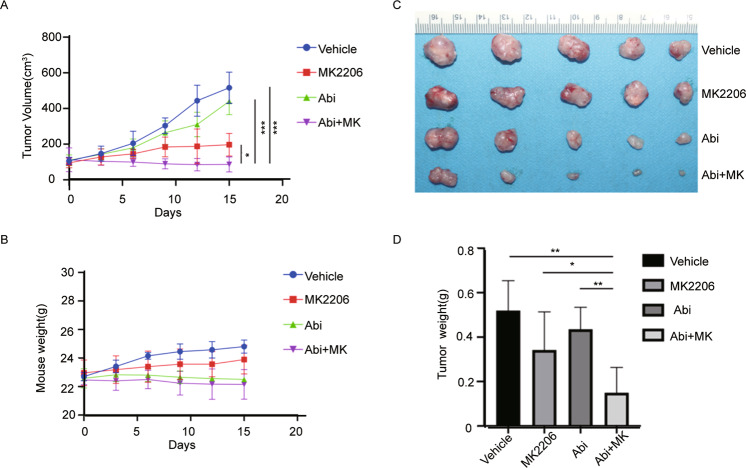
The combination of abiraterone and AKT inhibitor MK2206 synergistically inhibited the growth of PCa xenografts in vivo. CWR22Rv1 xenografts were established in the immune-deficient nude mice, and then treated with abiraterone or MK2206A alone or in combination for 15 days. **A** The growth curves of CWR22Rv1 xenografts. **B** The body weight of mice during drug treatment. **C** Tumor nodes were dissected and photoed at the endpoint of animal experiments. **D** The weight of tumor nodes at the endpoint of animal experiments. Data was represented as mean ± SD. *P < 0.05, **P < 0.01, ***P < 0.001.

We then validated the synergistic in vivo antitumor efficacy of abiraterone and MK2206. We established a subcutaneous xenograft tumor models in nude mice using CWR22Rv1 cells. Two weeks after tumor cell transplantation, the mice were administered by gavage with abiraterone or MK2206 alone or in combination. The gavages were administered every three days for continuous 15 days, the tumor volume and body weight were measured every 3 days to obtain tumor growth curves. The results showed that the combination of abiraterone and MK2206 inhibited the growth of tumor xenografts more significantly than abiraterone or MK2206 alone (Fig. 6A), but had not significant impact on the weight of mice (Fig. 6B). The tumor nodes were dissected at the endpoint of experiment, and the weight of tumor nodes verified the results of tumor growth curve (Fig. 6C and D). These results suggested that the combination of abiraterone and MK2206 showed superior anticancer efficacy to abiraterone or MK2206 alone, and the combination therapy is safe in the indicated dose window.

## Discussion

PI3k-AKT signaling pathway contributes to ADT resistance not only by activating its downstream signaling molecules such as RTKS but also by reversely regulating upstream molecules mTOR and AR [40]. In addition, it recently has been reported that 20% of high-grade PCa have the mutations of epigenetic regulators, which play driver roles for the development of mCRPC and drug resistance [41, 42].

UHRF1 is a critical epigenetic coordinator bridging DNA methylation and histone modifications by interacting with DNMT1 or HDAC1 individually [43], and aberrantly overexpresses in PCa specimens compared to non-malignant prostate tissues. UHRF1 steadily increases with the elevated degree of malignancy and the development of drug resistance [44, 45]. UHRF1 as a typical oncogene promoted PCa progression. knockdown of UHRF1 decreased the recruitment of DNMT1 to the CPG islands, thereby decreasing the maintenace of DNA methylation and re-elevating the epigenetic-silenced tumor suppressor genes such as RARB, CDH1, and PSP94. In addition, we recently reported that UHRF1 as AR co-activator regulates CDC6 gene transcription, and promotes anti-AR drug resistance by recruiting a demethyltransferase KDM4C [45].

It has been reported that the phosphorylation of UHRF1 protein is critical for its functions, UHRF1 Ser-661 may be phosphorylated by the CDK2-CCNA2 complex, thereby promoting zebrafish embryo development [39]. The phosphorylation of UHRF1 ser-298 facilitates ITG1 recognizing DNA replication initiation sites as well as histone H3K9me2/3 by altering the TTD domain of UHRF1 [46]. In addition, the phosphorylation of UHRF1 ser-652 reduces the interaction between UHRF1 and deubiquitinase USP7, which promotes the degradation of UHRF1 protein [38]. In the present study, AKT1 promoted UHRF1 protein stability by inducing UHRF1 phosphorylation at the threonine 210 residue, while the mutation of UHRF1 Thr-210 destroyed the stability of UHRF1 protein by inhibiting the protein interaction between UHRF1 with USP7, and promoting the protein interaction between UHRF1 and BTRC (Fig. 3H).

Furthermore, we found that targeting AKT1 by MK2206, a selective kinase inhibitor of AKT restored the sensitivity of abiraterone-refractory cells to abiraterone treatment. Moreover, the combination of two drugs significantly improved in vivo antitumor efficacy in PCa xenograft models (Fig. 6). Mechanistically, abiraterone plus MK2206 significantly decreased UHRF1 protein level, and elevated the epigenetic-silenced tumor suppressor genes such as P21, and decreased SYP and NCAM1, the typical NEPC markers. P21 is not only a tumor suppressor gene and cell-cycle inhibitor but also a marker of cellular senescence. The combinational therapy-elevated P21 overexpression resulted in cell-cycle arrest, and induced cellular senescence, and eventually triggered cell apoptosis (cleaved-PARP) (Fig. 2E and F.). In addition, it has been reported that PCa patients develops abiraterone resistance by promoting NEPC transdifferentiation [47]. The combinational therapy of two drugs remarkably decreased the levels of NCAM1 and SYP, suggesting that AKT inhibitor reversed the drug resistance of abiraterone by reversing the phenotype of NEPC.

Altogether, we for the first time revealed that AKT1 promoted drug resistance of PCa to abiraterone by regulating UHRF1 phosphorylation, thereby sustaining its protein stability. The aberrant overexpression of UHRF1 contributes to abiraterone resistance. Although UHRF1 inhibitor has not been commercially developed, the drug resistance of PCa to abiraterone may be reversed by inducing UHRF1 protein degradation with AKT inhibitor MK2206. This study provided a novel therapeutic modality that AKT1 inhibitor promoted drug sensitivity of abiraterone for mCRPC patients.

## Supplementary information

Supplementary methods

Supplementary figure legend

Supplementary Figure 1

Supplementary Figure 2

## Data Availability

The data used and analyzed in the present study are available in the article and its supplementary information. To view the data, visit 10.6084/m9.figshare.21258417.
