# Supplementary material for: AKT1 regulates UHRF1 protein stability and promotes the resistance to abiraterone in prostate cancer
Source: Oncogenesis. 2023 Jan 2;12(1):1. doi: 10.1038/s41389-022-00446-y (PMC9807647; doi:10.1038/s41389-022-00446-y)
Supplement: Supplementary file 1 — Supplementary methods [file 41389_2022_446_MOESM1_ESM.docx]

siRNA Transfection

HEK-293T, LNCaP-R, CWR22Rv1 and CWR22Rv1-R cells were seeded (2000 cells per well in 96-well plates, 1×10^5^ per well in 6 well plates and 1×10^6^ per dish in 6 cm dish), and the cells were transfected with NC siRNA or AKT1 siRNA mix (GenePharma, China) by TransIT-X2^®^ Dynamic Delivery System (Mirus Bio LLC, USA). The sequences of AKT1 siRNA and NC siRNA are as follows: AKT1-1 siRNA:5’ GGACAAGGACGGGCACAUUAATT3’, AKT1-2 siRNA:5’ CGCGUGACCAUGAACGAGUUUTT3’and NC siRNA: 5’UUCUCCGAACGUGUCACGUTT’.

Establishment of UHRF1 stable overexpressing cell lines

CWR22Rv1 were seeded in 6-well plates (1×10^5^cells per well), and were infected with lentivirus-UHRF1(Lenti-UHRF1) or lentivirus-empty vector(Lenti-EV) (OBiO Technology, Shanghai, China). The virus solutions were replaced by fresh media 12h after virus infection. The UHRF1-overexpressing or control cells were selected with 1ug/ml puromycin for 2 weeks.

Cell proliferation assay

CWR22Rv1-UHRF1 cells and CWR22Rv1-EV cells were seeded in 96-well plates(2000 cells per well) for 24h, and followed by transfection with AKT1 siRNA or NC siRNA. The cell viability was measured by CCK8 assays at different time points. Absorbance was measured at 450nm by VICTOR Nivo Multimode Plate Reader (PerkinElmer, USA)
