# Supplementary material for: AKT1 regulates UHRF1 protein stability and promotes the resistance to abiraterone in prostate cancer
Source: Oncogenesis. 2023 Jan 2;12(1):1. doi: 10.1038/s41389-022-00446-y (PMC9807647; doi:10.1038/s41389-022-00446-y)
Supplement: Supplementary file 2 — Supplementary figure legend [file 41389_2022_446_MOESM2_ESM.docx]

**Supplementary Figure legends**

**Supplement Figure 1. AKT1 knockdown promotes the degradation of UHRF1 protein through ubiquitin-proteasome pathway.** A and B. CWR22Rv1-R cells(A) or LNCaP-R cells(B) were were transfected with 40 pmol AKT1 siRNA for 48 h, and the protein levels of UHRF1 , AKT1 were assessed by western blotting. C and D. The mRNA levels of UHRF1 and AKT1 were measured by qRT-PCR. E. LNCaP which were transfected with AKT1 siRNA or NC siRNA were treated with 50 uM cycloheximide (CHX) for the indicated time, or were transfected with siRNA plus 50 uM CHX and 50 mM MG132 for 8 hours. UHRF1 protein levels were assessed by western blotting and the bands were quantified by image-J software. F. HEK-293T cells were co-transfected with the plasmids encoding His-UHRF1, Flag-myr-AKT1 and HA-ubiquitin for 24 hours. The cells were passaged into three dishes and cultured for 24 hours. Two of the dishes were transfected with NC siRNA or AKT1 siRNA for 48 hours, and then these cells were treated with 50 μM MG132 for additional 8 hours. UHRF1 protein was immunoprecipitated with ant-His antibody, and the ubiquitinated UHRF1 were assessed with anti-HA antibody. G, HEK-293T cells were co-transfected with the plasmids encoding His-UHRF1 or Flag-myr-AKT1. Then, the cells were passaged into three dishes and cultured for 24 hours, after then, two of the dishes were transfected with NC siRNA or AKT1 siRNA for 48 hours. UHRF1 protein was immunoprecipitated with anti-His antibody, and USP7 or BTRC were measured by western blotting. The presented results were representative of experiments repeated at least three times. Data was represented as mean ± SD. *P < 0.05, **P < 0.01, ***P < 0.001.

**Supplementary Figure 2** UHRF1 overexpression partially restored AKT1 knockdown-slowed cell proliferation.A, 2×10^3^ CWR22Rv1 cells stably expressing UHRF1(Lenti-UHRF1) or EV(Lenti-EV) were seeded into 96-well plates, and three time points were set for each cell type. On the next day, CWR22Rv1- Lenti-UHRF1 and CWR22Rv1- Lenti-EV were divided into two groups, and was transfected with NC siRNA or AKT1 siRNA, the cell proliferation rate were measured at 0h, 24h and 48h by CCK8 assay. The presented results were representative of experiments repeated at least three times. Data was represented as mean ± SD. *P < 0.05, **P < 0.01, ***P < 0.001.
