# Supplementary figures and images for: AKT1 regulates UHRF1 protein stability and promotes the resistance to abiraterone in prostate cancer
Source: Oncogenesis. 2023 Jan 2;12(1):1. doi: 10.1038/s41389-022-00446-y (PMC9807647; doi:10.1038/s41389-022-00446-y)

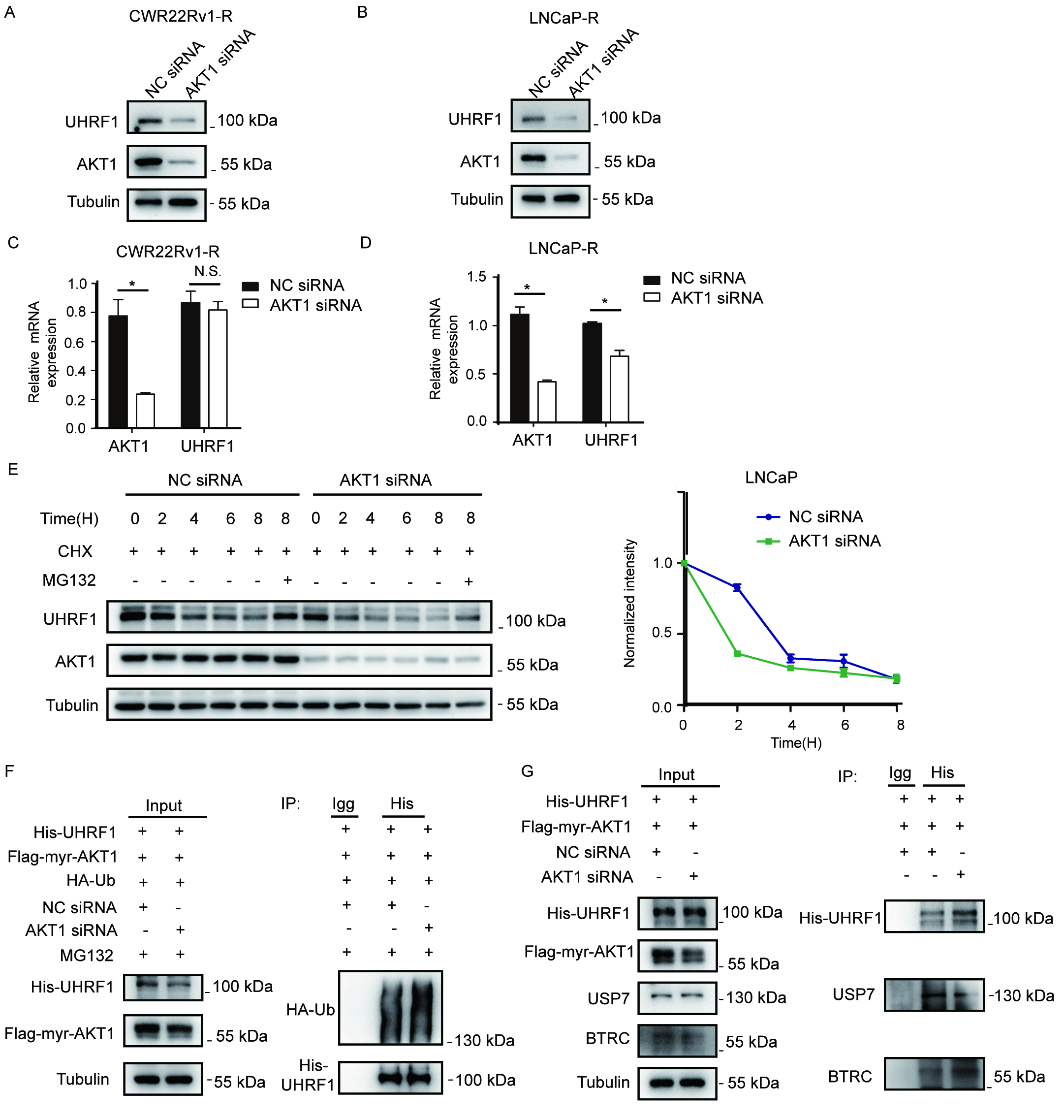

Supplement: Supplementary file 3 — Supplementary Figure 1 [file 41389_2022_446_MOESM3_ESM.tif]

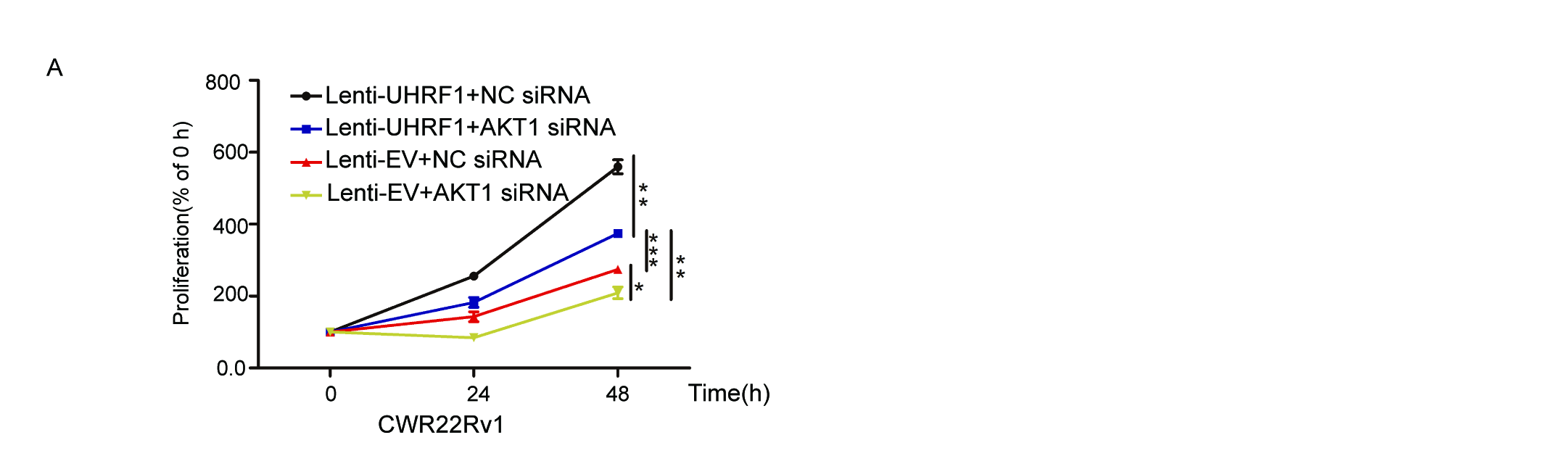

Supplement: Supplementary file 4 — Supplementary Figure 2 [file 41389_2022_446_MOESM4_ESM.tif]
